# Breast Cancer in a Caribbean Population in Transition: Design and Implementation of the Atabey Population-Based Case-Control Study of Women in the San Juan Metropolitan Area in Puerto Rico

**DOI:** 10.3390/ijerph17041333

**Published:** 2020-02-19

**Authors:** Rosa V. Rosario-Rosado, Cruz M. Nazario, Johan Hernández-Santiago, Michelle Schelske-Santos, Imar Mansilla-Rivera, Farah A. Ramírez-Marrero, Gilberto Ramos-Valencia, Consuelo Climent, Jing Nie, Jo L. Freudenheim

**Affiliations:** 1 Department of Biostatistics and Epidemiology, Graduate School of Public Health, Medical Sciences Campus, University of Puerto Rico, San Juan, PR 00936, USA; johan.hernandez@upr.edu (J.H.-S.); gilberto.ramos3@upr.edu (G.R.-V.); 2 Nutrition and Dietetics Program, Río Piedras Campus, University of Puerto Rico, San Juan, PR 00925-2537, USA; michelle.schelske@upr.edu; 3 Department of Environmental Health, Graduate School of Public Health, Medical Sciences Campus, University of Puerto Rico, San Juan, PR 00936, USA; imar.mansilla@upr.edu; 4 Department of Exercise Physiology, Río Piedras Campus, University of Puerto Rico, San Juan, PR 00925-2537, USA; farah.ramirez1@upr.edu; 5 Department of Pathology, School of Medicine, Medical Sciences Campus, University of Puerto Rico, San Juan, PR 00936, USA; consuelo.climent@upr.edu; 6 Department of Epidemiology and Environmental Health, University at Buffalo, State University of New York, Buffalo, NY 14214, USA; jingnie@buffalo.edu (J.N.); jfreuden@buffalo.edu (J.L.F.)

**Keywords:** breast cancer, Caribbean, epidemiology, Hispanic, life course, Puerto Rico

## Abstract

Global breast cancer incidence varies considerably, particularly in comparisons of low- and high-income countries; rates may vary even within regions. Breast cancer rates for Caribbean countries are generally lower than for North America and Europe. Rates in Puerto Rico are in the middle of the range between the highest and the lowest Caribbean countries. Populations in transition, with greater variability in risk factor exposures, provide an important opportunity to better understand breast cancer etiology and as potential sources of variation in rates. Understanding of exposures across the life span can potentially contribute to understanding regional differences in rates. We describe here the design and implementation of a population-based, case-control study in the San Juan Metropolitan Area (SJMA) of Puerto Rico, the Atabey Epidemiology of Breast Cancer Study. We describe steps taken to ensure that the study was culturally appropriate, leveraging the Atabey researchers’ understanding of the culture, local health system, and other required resources to effectively recruit participants. A standardized, in-person interview was developed, with attention to life course events customized to the study population. In order to understand variation in global breast cancer rates, studies customized to the populations outside of North America and Europe are required.

## 1. Introduction

Cancer of the breast is the most frequently reported cancer among women globally [1]. There is considerable variation in breast cancer incidence, with a more than a three-fold difference between lower- and higher-income countries [1]. Much remains unknown regarding the reason for these differences. While there is considerable information regarding risk factors for breast cancer in North American [2,3], European [4] and Asian populations [5], there is less known about risk factors in regions such as Africa [6,7] and Latin America, including the Caribbean [8,9,10]. Even within the Caribbean, age-standardized rates (ASR) for breast cancer incidence rates vary considerably between countries. Rates per 100,000 women are 22.0 in Haiti and 39.6 in Belize, while they are 94.7 in Barbados and 98.9 in the Bahamas [10]. The rate for Puerto Rico is intermediate, an incidence rate of 57.5 [10]. The ASR for breast cancer mortality also varies globally and within regions [10]. Among Caribbean nations, mortality rates per 100,000 range from 11.4 for French Guiana to 22.1 for Barbados and 26.3 for the Bahamas [10]; the rate is 13.0 for Puerto Rico [10].

Globally, many of the populations with lower breast cancer rates are in transition from a more traditional to a more westernized lifestyle, and are experiencing associated changes in breast cancer incidence and mortality over time [1,11]. Although breast cancer incidence rates are lower in Puerto Rico than in the USA [12,13], they are increasing in Puerto Rico while they are decreasing in the USA. Between 1987 and 2014, breast cancer incidence rates, age adjusted to the 2000 US standard population, increased 1.5% annually in Puerto Rico, from 60.5 to 88.5 per 100,000 women [12]. During the same time period in the USA, age-adjusted incidence rates declined from 134.5 to 130.6 [13]. On the other hand, age adjusted breast cancer mortality rates from 1987 to 2014 decreased in both Puerto Rico (19.0 to 18.6 per 100,000 women) and the USA (32.7 to 20.6 per 100,000 women) [14,15]. Populations in transition, such as Puerto Rico, provide an important opportunity for the evaluation of risk factors associated to breast cancer because of the variability in risk factors increasing the ability to examine the role of those risk factors in cancer etiology.

Although disparities in breast cancer incidence and prognosis by race, ethnicity and socioeconomic status in the USA have been documented [16], these differences have not been well studied among Hispanic women, including those living in Puerto Rico. Observed differences in breast cancer rates may reflect differences in risk factor prevalence as well as genetic factors, including those related to ancestry. The Puerto Rican genetic admixture includes pre-Columbian indigenous populations, European and African ancestry [17]. This Puerto Rican racial admixture may also provide insight to improve our understanding of differences in disease incidence among groups defined by race/ethnicity in the USA.

While there have been four analytical studies of breast cancer epidemiology in Puerto Rico [18,19,20,21], none were population-based. We describe here the design and characteristics of a population-based case-control study of breast cancer conducted among women living in Puerto Rico, the Atabey study (Atabey is a fertility goddess of the Taino native people in Puerto Rico). The methods and strategies used are described, including the development of a culturally sensitive and reliable instrument capable of eliciting recall from participants on a variety of variables related to lifestyle and environmental exposures. This study will allow us to understand better breast cancer rates and outcomes in Puerto Rico.

## 2. Materials and Methods

**Figure 1:**
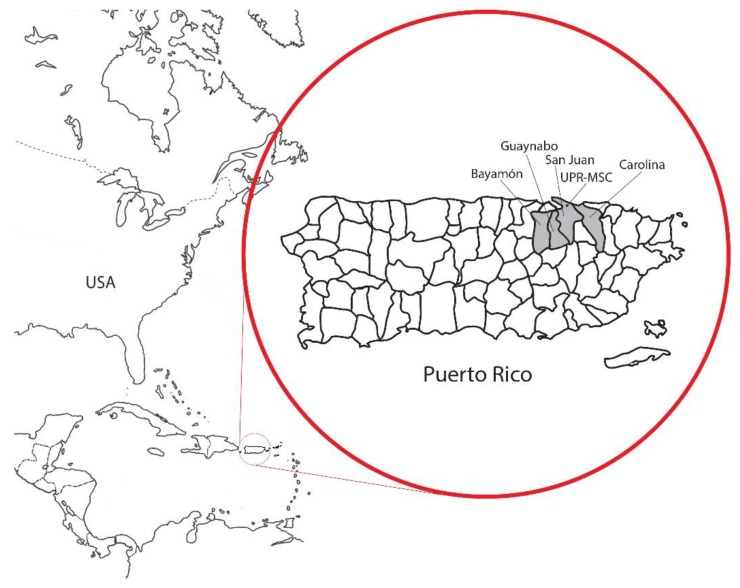
Geographical Location of Targeted Municipalities in the Atabey Study, Puerto Rico.

The Atabey Epidemiology of Breast Cancer Study is a population-based, case-control study focusing on the epidemiology of female breast cancer in Puerto Rico. This study is a collaboration between the University of Puerto Rico and the University at Buffalo, State University of New York. Study participants (30 to 79 years of age) were recruited between 2009 and 2014 from the Bayamón, Carolina, Guaynabo and San Juan municipalities in the San Juan Metropolitan Area (SJMA) (Figure 1). The study protocol was approved by the Institutional Review Board of the University of Puerto Rico Medical Sciences Campus (protocol numbers 0750108 and 0750117), the Institutional Review Board (IRB) of the University at Buffalo, CDMRP the sponsoring agency and all the participating clinical institutions. All participants provided informed consent.

### 2.1. Study Infrastructure

Because a population-based study of breast cancer had not previously been conducted in Puerto Rico, the study required the development of a research infrastructure as well as the maximization of existing resources. The knowledge and cultural competency of the Puerto Rico-based investigators was critical to the study development and implementation. Existing relationships with administrative and medical personnel, skilled potential staff, combined with understanding of local resources, local hospital procedures and local IRB requirements were essential for the effective implementation of the study.

The study leveraged several important existing resources. These included the Puerto Rico Cancer Registry, which was used to assist in case ascertainment, the Puerto Rico Health Interview Survey (PRHIS), which was used as the model for the control ascertainment, and the Puerto Rico Clinical and Translational Research Consortium (PRCTRC), a University of Puerto Rico Medical Science Center facility where the blood draw, anthropometry and interviews were conducted, all described in detail below.

The expertise of the local investigators was significant in hiring and training of study personnel. Their knowledge permitted the recruitment of interviewers and a Community Outreach (CO) worker who were able both to understand the study requirements and to interact appropriately with participants and potential participants to obtain the highest quality and most complete data. Study participants valued the fact that the Puerto Rican research was conducted by local investigators.

### 2.2. Case Ascertainment and Recruitment Procedures

**Figure 2:**
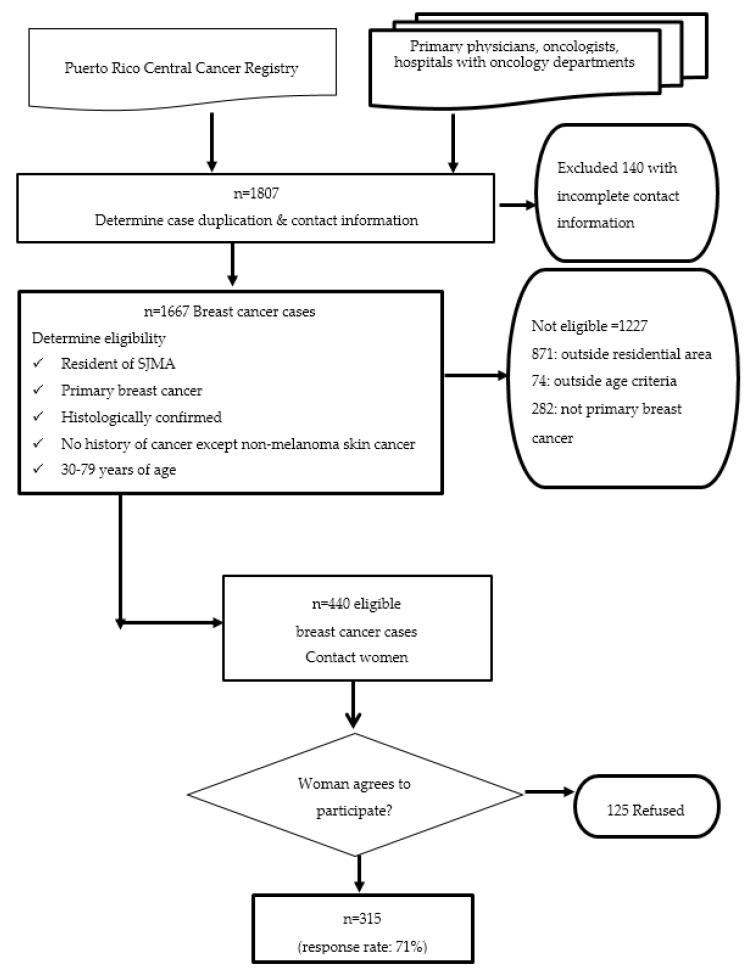
Recruitment of Breast Cancer Cases in the Atabey Study, San Juan Metropolitan Area (SJMA), Puerto Rico.

Cases were women, 30 to 79 years of age, residents of the SJMA, with incident, primary, pathologically confirmed breast cancer and no history of previous cancer other than non-melanoma skin cancer. They were ascertained from the hospitals and clinics where breast cancer patients were diagnosed or treated: hospitals with oncology departments, cancer registries and private oncologist’s offices within the SJMA. A list of hospitals and clinics that report breast cancer cases was obtained from the Puerto Rico Central Cancer Registry (PRCCR). The PRCCR data were also used as a cross check to identify any potential cases who had not been ascertained from the other sources. The PRCCR gathers cancer data through an electronic reporting system and is a population-based registry that achieves 95% completeness of data collection from primary sources (i.e., hospitals, physicians’ offices and pathology laboratories), with 92.8% of cancer cases having histologic confirmation of disease [22]. The reporting of cancer cases to the registry is mandated by law. Even though the PRCCR is the definitive source of breast cancer case information, reporting of cancer cases may be delayed. Therefore, cases were primarily ascertained from referring oncologists for more rapid ascertainment (Figure 2). Once the contact information was determined, potential participants were called to explain the Atabey study and to invite them to participate. Once they agreed to participate, a unique identifying code number was assigned for tracking cases. An interview was scheduled and all data and samples, including questionnaire and anthropometry data, and biological samples as well as pathology data from tumors were collected.

### 2.3. Control Ascertainment and Recruitment Procedures

Controls were women aged 30–79, with no history of cancer, other than non-melanoma skin cancer, who were residents of the SJMA. They were selected and frequency-matched to cases by broad geographical residential area (municipality or county). The number of potential controls required for each municipality was based on the expected number of new breast cancer cases by municipality, informed by the PRCCR data.

The sampling frame and the strategy for the selection of population-based controls was provided by Dr. Gilberto Ramos Valencia, Director of the Puerto Rico Health Interview Survey (PRHIS), a national cross-sectional health survey with a multistage sampling procedure of geo-political conglomerates (municipalities or counties and census blocks) as determined by the USA Census from the year 2000 [23]. The selection of potential controls for the Atabey study was based on a multistage cluster sample stratified by targeted municipalities. The sampling frame consisted of census blocks as primary sampling units and household segments as secondary sampling units.

**Figure 3:**
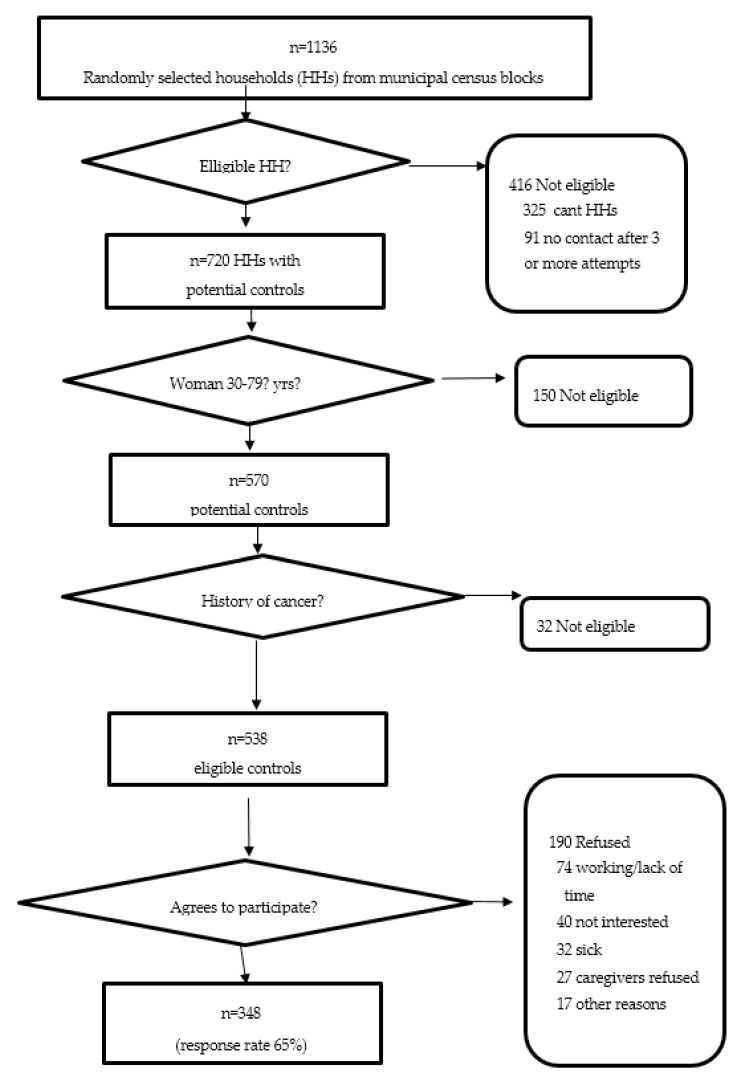
Selection of population-based controls in the Atabey Study, Puerto Rico.

Census blocks were randomly selected from the municipalities of San Juan, Carolina, Bayamon and Guaynabo (San Juan Metropolitan Area, SJMA). The number of households in each selected census block was calculated and each household was considered a random point. The number of expected breast cancer cases from the municipalities in the target area (SJMA) was used to estimate the number of households to select in each block. From the randomly selected blocks, those that had the largest number of households were prioritized. The process was repeated until the total number of households needed to complete the identification of the expected number of controls from each municipality was achieved. The total number of households to be visited was calculated taking into consideration an expected refusal rate of 25%. The module “EpiTable” from EpiInfo 6, version 6.04d [24] was used to randomly organize the order of random points to be visited by the CO worker (Figure 3).

The CO was trained to apply a systematic recruitment approach to reduce selection bias and to recruit a representative sample of population controls. On a weekly basis, the project coordinator met with the CO to discuss and plan the identification and recruitment of potential controls in the selected blocks. The CO received the precise address and description of the selected block (county, census track and census block), the address of the initial random point and the number of controls to recruit. The CO could not substitute the selected blocks or the randomly selected initial household assigned to the block.

Once the initial random household was identified in the selected block, the CO visited the first household to recruit eligible controls. At each household, the CO obtained information regarding potential and eligible controls within the household. The CO asked if there was a woman living in the home who was between 30 and 79 years of age. If affirmative, the Atabey Study was described, and eligibility was confirmed. If an eligible woman was present, she was invited to participate. If more than one eligible woman lived in the same household, the CO invited the oldest eligible woman first. If she refused to participate then the CO could invite the second oldest woman.

If there were no adults present in the household, a flyer describing the Atabey Study was left. The CO re-visited a household up to three times to determine if there were eligible controls in the household. The CO then visited the next house to the left, taking into consideration the number of potential controls to be recruited from that block. If the number of potential controls in the block was completed, the CO would visit the next assigned block, following the same process as previously described. A summary of the strategies used for the control identification and the recruitment process is shown in Figure 3.

Once they agreed to participate, controls were assigned a unique identifying code number for tracking, an interview was scheduled and all data and samples, including a questionnaire and anthropometry data, and biological samples were collected.

### 2.4. Questionnaire Development

Careful attention to the Puerto Rican population and culture was woven into the questionnaire development. The instrument incorporated questions about known breast cancer risk factors, including sections on demographics, occupation, residential history, early life exposures, usual diet anchored to the year prior to the interview (including consumption of traditional Puerto Rican foods), adolescent diet, reproductive history, weight history, smoking history, exposure to environmental tobacco smoke, physical activity history, sun exposure history, personal and family history of diseases, environmental and occupational exposures and vitamin and medication use.

While much of the questionnaire was similar to others used in other epidemiologic studies of breast cancer, many important culturally-sensitive differences were incorporated. For example, researchers discussed at length the acceptability of particular questions or procedures before including them. In particular, there was discussion regarding taking anthropometric measures and measuring skin pigmentation using colorimetry, as well as the development of questions regarding exposures that might help characterize transition among risk factors. All questionnaire and interviewer-related materials were translated to English for complete review by the investigative team at the University of Buffalo and then back translated to Spanish.

To enhance cultural sensitivity, all staff interacting with study participants were local women with research experience and who spoke regional Spanish. They were trained extensively regarding study procedures. Training focused on prevention of bias in interactions with participants, on making participants feel comfortable and listening to them, but following the study protocol to keep the interview on track. During training, interviewers and investigators did role playing exercises to simulate appropriate conduct during the interview. Both nurses, who drew blood, did anthropometric and colorimetry measures, and interviewers were blinded to case-control status and to the study hypotheses. Quality control and re-training were conducted periodically to ensure compliance with study protocols by nurses and interviewers.

To promote and solidify collaborative ties, the study investigators met by teleconference weekly to discuss the study development, implementation and data analyses. In addition, study investigators from the University of Puerto Rico travelled to Buffalo and University of Buffalo investigators travelled to Puerto Rico. The frequent communication was vital in the development of a strong collaboration, for sharing of expertise and for ongoing problem-solving of issues as they arose.

### 2.5. Data Collection

Study nurses collected biological samples from each participant as well as anthropometric and skin pigmentation measurements following a detailed, standardized protocol. Fasting blood (27 mL of blood) was collected from study participants. Serum, plasma, buffy coat and red blood cells were aliquoted to 0.5 mL straws and stored in −80 °C freezers. Each biological sample was marked by a unique identifier using scannable bar codes. For those few participants who refused or were unable to provide a blood sample, a saliva sample was obtained for DNA extraction. All biological samples (blood or saliva) were processed at the PRCTRC. Frozen straws were shipped overnight on dry ice to Buffalo, NY, for long-term specimen storage at the University at Buffalo; participant samples were divided into multiple shipments to avoid the possibility of total sample loss due to an unforeseen event. The Puerto Rican laboratory technician was trained in the processing and aliquoting protocol at the biospecimens laboratory at Roswell Park Comprehensive Cancer Institute in Buffalo, New York.

Interviews were conducted in-person by trained personnel using a structured questionnaire. As noted above, the questionnaire was constructed specifically for this study. It utilized some questions regarding breast cancer risk factors based on questionnaires from other, similar studies, adapted to the particular needs of this population as well as other questions specific to Puerto Rican women. The data collection instrument included questions regarding demographic, occupation, residential history, early life exposures, reproductive factors, diet (usual diet during adolescence and adulthood), vitamin use, sun exposure and medical history including use of some medications. At the end of the interview, once data relevant to both cases and controls were obtained, additional clinical information was collected from the breast cancer cases. These data included information regarding breast cancer treatment (breast biopsies, surgery, radiation therapy and chemotherapy). Interviewers were blinded to case-control status up until questions from this section of the questionnaire were asked, when knowledge of case control status was required.

Breast carcinoma is a heterogeneous disease comprised of different histopathological and clinical features. Classical immunohistochemical (IHC) markers such as estrogen receptors (ER), progesterone receptors (PR) and human epidermal growth factor receptor 2 (Her2) have important therapeutic prognostic implications.

Study researchers obtained information on breast carcinoma pathology from hospital medical records or the Puerto Rico Central Cancer Registry. One investigator (CC), reviewed and cross-checked pathology data following the system described by Howlader et al. [25]. Breast carcinoma cases were classified according to estrogen (ER) and progesterone (PR) and HER2 receptor status. Cases coded as borderline were re-coded using information from the pathology report [25,26]. When Her2 was equivocal and FISH information was available, it was used to recode the case. Breast tumors were classified according to subtypes [26,27,28,29].

## 3. Results

As shown in Figure 2, study collaborators provided 1807 referrals of potential breast cancer cases; of those, the contact information was either incomplete, missing or incorrect for 140. Of the 1667 potential participants, 1227 breast cancer cases did not meet study inclusion criteria: 871 lived outside the targeted geographical region; 282 did not meet inclusion criteria based on their pathology reports (date of diagnosis not within the study time period, not a primary breast cancer tumor or pathology not conclusive for invasive carcinoma); 74 were ineligible for other reasons, such as age. There were 440 breast cancer cases who met the eligibility requirements: primary, incident, primary pathologically confirmed breast cancer with no history of previous cancer, aged 30 to 79, and living in San Juan, Bayamón, Guaynabo or Carolina municipalities in the SJMA and diagnosed within the study time period (2009 to 2014). Of those, 315 (71.3%) agreed to participate and completed all study procedures.

As shown in Figure 3, during the recruitment of controls, 1136 households were visited; 416 houses were either vacant or contact with an informant could not be completed after three or more attempts. In households where a contact was made, 720 women were contacted. Of these, 150 were not eligible to participate because of age, and 32 had a previous history of cancer. Of the 538 eligible controls in the study, 348 (64.7%) agreed to participate and completed the interview and clinical evaluation.

In total, in this population-based study, there were 315 cases and 348 controls who met eligibility requirements, completed the interview and provided biological samples. There were 41 participants for whom a saliva sample was obtained; the remainder provided a blood specimen. Pathological data were obtained from all breast cancer cases from hospital records or the Puerto Rico Central Cancer Registry.

Characteristics of Atabey participants are shown in Table 1. Breast cancer cases [mean 58.7 years] were somewhat older than controls [mean 54.0 years). Cases had more education: 84.8% had 12 years or more of schooling, compared to 75.9% of controls. Cases [mean 30.0 kg/m^2^) had lower body mass index (BMI) than controls [mean 31.2 kg/m^2^].

With regard to reproductive factors, cases and controls were similar in age at menarche (mean 12.2 and 12.5 years of age, respectively). Cases were more likely to be nulliparous than controls (17.5%, 14.1%, respectively). Among parous participants, the mean number of births was somewhat higher for controls (2.9) than for cases (2.6). Among parous participants, the average age at first birth was higher for cases (24.3 years) than controls (22.8 years). Among post-menopausal participants, the age at menopause was 47.4 years for cases and 46.8 years for controls.

Breast cancer cases had a higher likelihood of having a family history of breast cancer and a personal history of benign breast disease. Family history of breast cancer was reported by 21.0% of breast cancer cases and 8.6% of controls; 46.7% of cases and 25.6% of controls had a previous benign breast disease.

Characteristics of the cases’ tumors are shown in Table 2. Estrogen receptor (ER) status was known for 76.2%, progesterone receptor (PR) status for 74.3% and HER2 status for 59.4%. Of those with pathology test results, 78.3% were ER+, 67.9% were PR+ and 20.3% were Her2 positive; 66.2% were ER+/PR+ and 20.5% were ER-/PR-. The vast majority of cases (63.2%) with data were classified as Luminal A (ER+, PR+, Her2-). After excluding those without receptor data, the distribution of Luminal A breast cancer cases was similar to other populations [30].

## 4. Discussion

To our knowledge, this is the first population-based, case-control study of incident breast cancer conducted in Puerto Rico. Previous studies in Puerto Rico focused on molecular factors associated with DNA repair among breast cancer women in Puerto Rico [19,20,21]. One other study evaluated risk factors using incident breast cancer cases from a clinic population; the study was no population-based in that the controls were selected from those with negative mammograms at that clinic [18].

The Atabey study represents a long-lasting collaboration between the University of Puerto Rico (a minority institution) and the University at Buffalo (a recognized research institution) and Roswell Park Comprehensive Cancer Center (recognized for cancer research). Intercampus collaboration between the UPR at Río Piedras and UPR Medical Sciences Campus was obtained.

There have been important lifestyle changes for known or suspected breast cancer risk factors in Puerto Rico. For example, median years of education of females in Puerto Rico increased from 3.3 school years in 1950 to 9.2 school years in 1980 [31]. Fertility patterns have also changed. Among women aged 15–44 years in Puerto Rico, there were 173.4 births annually per 1000 women of reproductive age in 1930–1939 [31], decreasing to 41.8 in 2016 [32]. The fertility rate among young women (15–19 years of age) in particular has dropped; from 72.5 births per 1000 women aged 15 to 19 in 2000 to 29.7 in 2016 [32]. The changing incidence is likely related to changes in the population in education, breast feeding, physical activity and obesity [10,11,33]. This study provides the opportunity to examine association of these risk factors that are in flux with breast cancer incidence. Systematic reviews [10,11] in the Caribbean and Latin America as well as Cazap study [33] have discussed issues regarding changing incidence patterns and its association with the characteristics of populations in transition. Most pertinent characteristics were education, breast feeding, physical activity and obesity [10,11,33].

Studies of populations in transition provide the opportunity to examine factors related to changes in rates of cancer incidence and mortality. With greater variability in exposures, it may be possible to identify associations that are more difficult to determine in populations with greater uniformity in exposures. Furthermore, studies such as this one are important in capacity building, providing experience in research to all of those involved in the study design, implementation and analysis; further the study results are an important potential resource to other researchers and for training.

### 4.1. Study Limitations

The challenges of the study were considerable. Population-based studies such as this one with strict eligibility criteria impose an enormous burden. Without any population-based listing of the SJMA, the selection of appropriate population-based controls required a probabilistic sampling combined with verification of eligible households in the field. Having an experienced study coordinator and a CO with familiarity regarding the region were fundamental to the project. Case ascertainment required identification and enlisting cooperation of those providing care to breast cancer cases, as well as engaging administrators, other clinicians and institution-specific IRBs. While study participation was high, contacting and enrolling participants was challenging. For each identified participant, six to eight telephone calls were made to schedule an interview, remind them of their appointment, reschedule appointments and motivate them to participate. While response rates for both cases and controls were good relative to other case-control studies, there remains the possibility of selection bias, that is, that those participating differed systematically from those who did not participate and that those differences were not the same for cases and controls. Furthermore, as for any case-control study, there is the potential for recall bias. Every effort was made to reduce the likelihood of such biases in report by training the interviewers in the administration of the structured interview, providing context to the participants to assist them in recalling the past. There is evidence that recall bias in case control studies may be modest [34,35]. Finally, there is likely non-differential error in the report of exposures; such error would bias results toward the null.

### 4.2. Study Strengths

The population-based study design is a strength of this study. Furthermore, the relatively high response rates provide for external validity. The development of the study protocol and questionnaire, as well as the training of the staff, was tailored to the Puerto Rican culture and critical to the study’s success. Measurement and data collection procedures were standardized to minimize recall and interviewer bias. Interviewers were trained to follow standardized procedures and were of the same ethnicity as the Atabey study participants. Interviewers used visual aids to improve recall and minimize measurement error.

## 5. Conclusions

This is the first population-based, case-control study of breast cancer conducted in Puerto Rico. Data were collected through a standardized in-person interview with special attention to life course events. This focus was novel for the study of breast cancer in a population with lower rates that is in transition. These results have the potential to benefit breast cancer prevention among other Hispanic women living in the U.S. who comprise nearly 16.2% of the U.S. female population [36] and share common genetic admixture and lifestyle risk factors with Puerto Rican women living in the Island, thus, reducing U.S.-based breast cancer disparities. In addition, findings from this study could provide insight regarding breast cancer disparities more generally. The study was culturally appropriate, conducted by researchers who were part of the culture, who had in-depth knowledge of the local health system, could identify appropriate contacts within that system, and were knowledgeable with regard to strategies for successful recruitment and data collection from participants.

Studies of populations in transition provide the opportunity to examine factors related to changes in rates of cancer incidence and mortality. With greater variability in exposures, it may be possible to identify associations that are more difficult to determine in populations with greater uniformity in exposures. In addition, recall of early life factors may be more possible with larger variation in exposures, with participants being able to recall at minimum whether they had or had not been exposed for example to foods part of a traditional diet. Furthermore, studies such as this one are important in capacity building, providing experience in research to all of those involved in the study design, implementation and analysis with study resources an important potential resource to other researchers and for training.

## Figures and Tables

**Table 1:** Characteristics of Atabey Study Participants by Case-Control Status (2009–2014).

Participant Characteristics	Cases (*n* = 315)	Controls (*n* = 348)
	Mean (SD)	Mean (SD)
Age (yrs)	58.7 (11.0)	54.0 (13.4)
Body Mass Index (BMI)	30.0 (6.0)	31.2 (7.4)
Age at menarche (yrs)	12.2 (1.6)	12.5 (1.8)
Number of births *	2.6 (1.3)	2.9 (1.4)
Age at 1st birth *	24.3 (5.0)	22.8 (5.4)
Age at menopause (yrs) **	47.4 (6.9)	46.8 (6.4)
	*n* (%)	*n* (%)
Education		
<12 yrs	48 (15.2)	84 (24.1)
>12 yrs	267 (84.8)	264 (75.9)
Parity		
Nulliparous	55 (17.5)	49 (14.1)
Parous	260 (82.5)	299 (85.9)
Menopausal Status		
Pre-	84 (26.7)	134 (38.5)
Post-	231 (73.3)	214 (61.5)
Family History of Breast Cancer (yes)	66 (21.0)	30 (8.6)
History of Benign Breast Disease (yes)	147 (46.7)	89 (25.6)

* among parous only; ** among postmenopausal only.

**Table 2:** Breast cancer types and subtypes in the Atabey study.

Tumor Receptor Status				Cases (*n* = 315)	Percentage	Percentage Excluding Unknowns
Estrogen Receptor (ER) Status						
ER-				52	16.5	21.7
ER+				188	59.7	78.3
Unknown				75	23.8	
Progesterone Receptor (PR) Status						
PR-				75	23.8	32.1
PR+				159	50.5	67.9
Unknown				81	25.7	
Her2 Status						
Her2-				149	47.3	79.7
Her2+				38	12.1	20.3
Unknown				128	40.6	
Hormone Receptor (HR) Status						
ER+ & PR+ (HR positive)				155	49.2	66.3
ER- & PR- (HR negative)				48	15.2	20.5
ER+ & PR- (HR positive)				27	8.6	11.5
ER- & PR+ (HR positive)				4	1.3	1.7
Unknown				81	25.7	
Subtypes	ER	PR	Her2			
Luminal A	+	+	-	117	37.1	63.2
Luminal B	+	+	+	27	8.6	14.6
Her2-enriched	-	-	+	11	3.5	5.9
Triple negative	-	-	-	30	9.5	16.2
Unknown				130	41.3	
